# SIRT2 Is Critical for Sheep Oocyte Maturation through Regulating Function of Surrounding Granulosa Cells

**DOI:** 10.3390/ijms23095013

**Published:** 2022-04-30

**Authors:** Xiaohuan Fang, Wei Xia, Sa Li, Yatian Qi, Mingzhi Liu, Yang Yu, Hanxing Li, Mengqi Li, Chenyu Tao, Zhigang Wang, Junjie Li

**Affiliations:** 1 College of Animal Science and Technology, Hebei Agricultural University, Baoding 071000, China; fangxiaohuan94@163.com (X.F.); xiaweihawaii@163.com (W.X.); lisa1994157@163.com (S.L.); qiyatian187@163.com (Y.Q.); rankking2022@163.com (M.L.); yyang980828@163.com (Y.Y.); lihanxing1999@163.com (H.L.); lmq110119112@163.com (M.L.); taochenyuty@163.com (C.T.); kjjw@hebau.edu.cn (Z.W.); 2 Research Center of Cattle and Sheep Embryo Engineering Technique of Hebei Province, Baoding 071000, China

**Keywords:** SIRT2, sheep granulosa cells, oocytes in vitro maturation, mitochondria, mitophagy

## Abstract

Oocyte in vitro maturation is crucial for in vitro embryo production technology, which provides oocytes resources for in vitro fertilization and somatic cell nuclear transfer. Previous studies proved that SIRT2, a member of the sirtuin family, plays a role in oocyte meiosis, but its role in sheep oocyte maturation and its regulating mechanism remains unknown. Firstly, we confirmed the role of Sirt2 in sheep oocytes maturation by supplementation of SIRT2 inhibitor and activator. To further explore the specific mechanism, we performed knockdown of Sirt2 in granulosa cells and then cocultured them with oocytes. Moreover, we determined the effects of Sirt2 on granulosa cell oxidative apoptosis, cell migration, and diffusion, and examined its effects on granulosa cell mitochondrial function, mitophagy, and steroid hormone levels. The results showed that supplementation of SIRT2 inhibitor decreased the oocytes maturation rate (69.28% ± 1.28 vs. 45.74% ± 4.74, *p* < 0.05), while resveratrol, a SIRT2 activator, increased its maturation rate (67.44% ± 1.68 vs. 78.52 ± 1.28, *p* < 0.05). Knockdown of Sirt2 in sheep granulosa cells also reduced the oocytes maturation rate (47.98% ± 1.43 vs. 33.60% ± 1.77, *p* < 0.05), and led to decreased cell migration and expansion ability, oxidative apoptosis, abnormal mitochondrial gene expression, decreased mitochondrial membrane potential and ATP level, and increased mitophagy level. Overexpression of Sirt2 improved mitochondrial membrane potential and ATP level and improved mitochondrial function. Furthermore, we found that Sirt2 knockdown in granulosa cells promotes the secretion of P_4_ through regulating p-ERK1/2. In conclusion the present study showed that SIRT2 is critical for sheep oocyte maturation through regulating the function of ovarian granulosa cells, especially affecting its mitochondrial function.

## 1. Introduction

The granulosa cell is one of the most important components of mammalian follicle structure, which coexists in the same microenvironment with oocyte and interacts with it to regulate its growth and maturation [1]. Granulosa cells supply amino acids, glutathione, glucose metabolites, and other nutrients to oocytes through direct cell-to-cell communication or paracrine secretion to provide energy for oocytes [2,3,4].

Mitochondria of granulosa cells are the most crucial organelles, and have been shown to play a central role in maintaining adequate oocyte ATP levels by providing metabolic support through gap junctional communication [5]. If mitochondria are damaged, excessive opening of permeability conversion pores on the mitochondrial membrane will reduce the level of mitochondrial membrane potential, leading to mitochondrial swelling and a decrease in ATP level [6]. The ATP level in granulosa cells is closely related to the growth and development of oocytes. It was also found that the ATP level in oocytes surrounding granulosa cells was significantly higher than that in denuded oocytes (DOs) during maturation [7]. In addition to mitochondrial oxidation for energy, granulosa cells can also affect oocyte development by regulating the secretion of steroid hormones. It has been proved that granulosa cells can secrete more estradiol (E_2_) and less progesterone (P_4_) to promote oocyte maturation [8].

The sirtuin family is an important class of histone deacetylases. SIRT2 is a member of the sirtuin family and participates in regulation of metabolism, inflammatory signals, oxidative stress, and other physiological processes [9,10,11]. In recent years, studies have found that SIRT2 can affect the development of female follicles and oocyte maturation through a variety of physiological regulation, such as oxidative stress and energy metabolism [12,13]. In addition, studies indicated that SIRT2 is closely related to extracellular signal-regulated kinase 1/2 (ERK1/2) signaling pathway, which can inhibit the phosphorylation of connexin 43 by downregulating ERK and promoting information exchange between cells [14]. ERK1/2, as an important signaling pathway kinase, is involved in the secretion of steroid hormones, in which activation of ERK1/2 enhances SF-1 phosphorylation and StAR gene transcription, thereby promoting steroid synthesis [15].

At present, the role of SIRT2 in sheep granulosa cells on oocyte maturation and its mechanism remain to be determined. Therefore, the aim of this study was to investigate the effects of SIRT2 in sheep granulosa cells on the migration and diffusion, mitochondrial function, and steroid hormone secretion of granulosa cells, so as to improve the in vitro maturation efficiency of sheep oocytes.

## 2. Results

### 2.1. Inhibition or Activation of SIRT2 Affects Sheep Oocytes In Vitro Maturation

**Figure 1:**
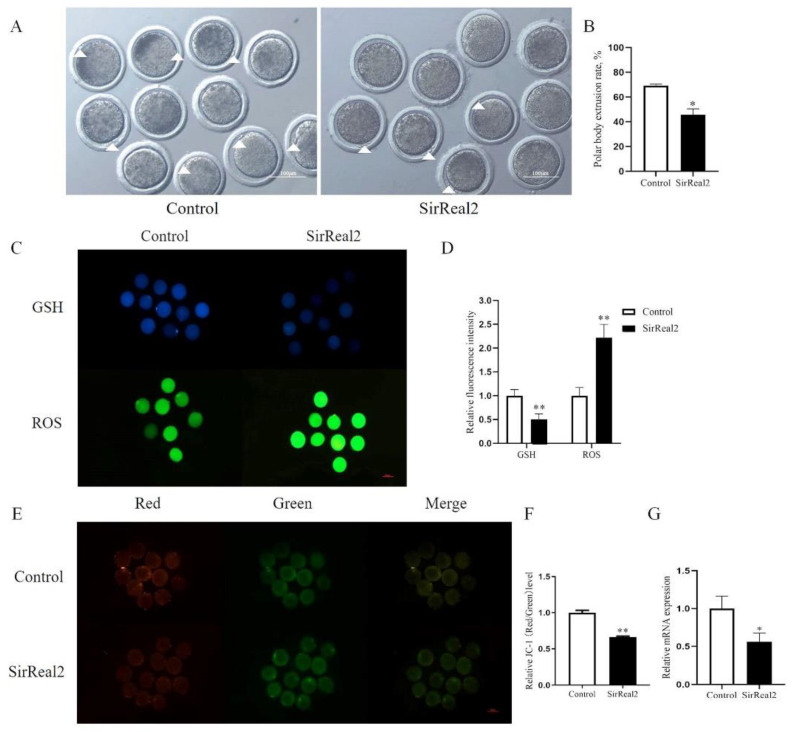
Inhibition of SIRT2 in oocytes affects maturation rate, GSH, ROS, and mitochondrial membrane potential. (**A**) Representative images of oocyte maturation after Sirt2 inhibition. The white triangle represents the extrusion of the polar body. Bar = 100 μm. (**B**) The maturation rate after inhibition of SIRT2 in oocytes. (**C**) Representative images of GSH and ROS after SIRT2 inhibition in oocytes. Bar = 100 μm. (**D**) The relative levels of GSH and ROS in oocytes after SIRT2 was inhibited. (**E**) Red and green fluorescent images of JC-1 staining after inhibiting SIRT2 in oocytes. Bar = 100 μm. (**F**) The relative level of JC-1 after inhibiting SIRT2 in oocytes. (**G**) Sirt2 mRNA level after Sirt2 inhibition. The graph shows the mean ± SEM of the results obtained in three independent experiments. *, significant difference (*p* < 0.05). **, significant difference (*p* < 0.01).

To firstly determine the effect of SIRT2 on sheep oocyte maturation, we inhibited SIRT2 by treating with SirRreal2, a SIRT2 inhibitor in COCs, and checked its oocyte in vitro maturation rate. The maturation rate of sheep oocytes was significantly reduced after 24 h treatment with 5 μM SirRreal2 (69.28% ± 1.28 vs. 45.74% ± 4.74, *p* < 0.05, Figure 1A,B). In addition, ROS levels were significantly increased in the SIRT2-inhibited oocytes, while GSH and mitochondrial membrane potential levels were significantly decreased (*p* < 0.01, Figure 1C–F). Moreover, we proved that Sirt2 mRNA level decreased significantly in the inhibition group.

**Figure 2:**
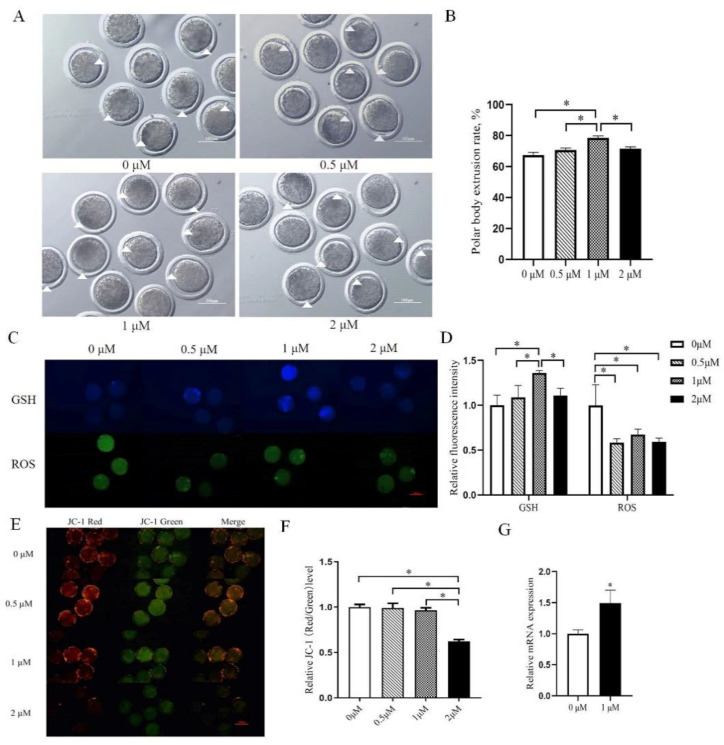
Resveratrol affects maturation rate, GSH, ROS, and mitochondrial membrane potential in oocytes. (**A**) Representative images of oocyte maturation after resveratrol treatment. The white triangle represents the extrusion of the polar body. Bar = 100 μm. (**B**) The maturation rate after treatment with different concentrations of resveratrol in oocytes. (**C**) Representative images of GSH and ROS after treatment with different concentrations of resveratrol in oocytes. Bar = 100 μm. (**D**) The relative levels of GSH and ROS in oocytes after treatment with different concentrations of resveratrol. (**E**) Red and green fluorescent images of JC-1 staining after treatment with different concentrations of resveratrol in oocytes. Bar = 100 μm. (**F**) The relative level of JC-1 after treatment with different concentrations of resveratrol in oocytes. (**G**) Sirt2 mRNA level after 1μM resveratrol treatment. The graph shows the mean ± SEM of the results obtained in three independent experiments. *, significant difference (*p* < 0.05).

Further, COCs were then placed into a mature solution containing resveratrol (0, 0.5, 1, 2 μM), an activator of SIRT2, for in vitro maturation. Compared with control, the oocyte maturation rate (67.44% ± 1.68 vs. 78.52 ± 1.28) and GSH level in 1 μM resveratrol supplemental group increased significantly (*p* < 0.05, Figure 2A–D), the ROS level was significantly decreased (*p* < 0.05, Figure 2C,D), and no difference was found in the level of mitochondrial membrane potential (*p* > 0.05, Figure 2E,F). In addition, the Sirt2 mRNA level was significantly increased after 1 μM resveratrol treatment. These inhibition and activation results indicate that SIRT2 can affect sheep oocytes maturation and disturb its oxidative level.

### 2.2. Sirt2 Is Critical for Sheep Oocytes In Vitro Maturation by Affecting the Apoptosis, Expansion, and Secretion of Granulosa Cells

**Figure 3:**
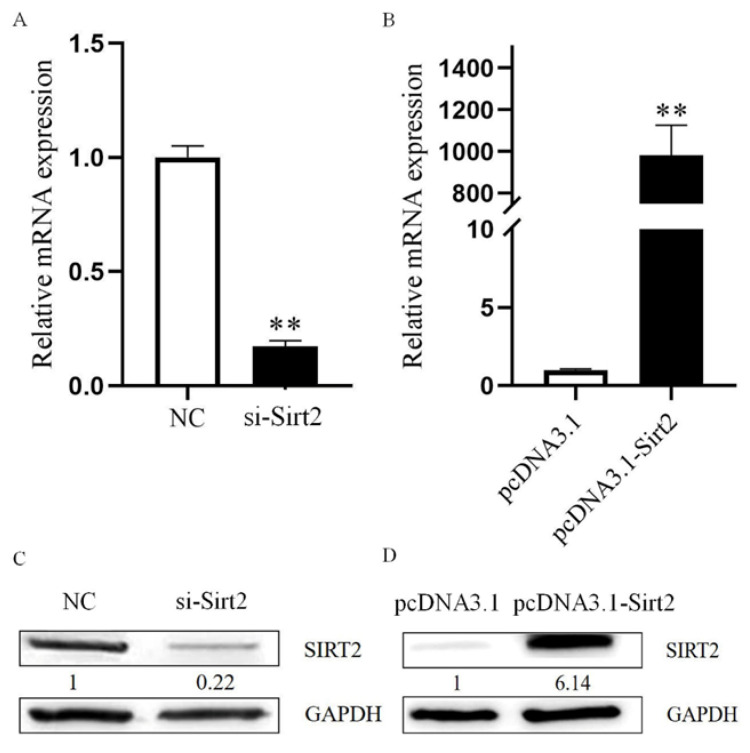
Determination of Sirt2 knockdown and overexpression efficiency in present study. (**A**) Detection of mRNA level of Sirt2 by qRT-PCR after siRNA knockdown. (**B**) Detection of mRNA level of Sirt2 by qRT-PCR after overexpression. (**C**) Detection of protein level of SIRT2 by Western blot after siRNA knockdown. (**D**) Detection of protein level of SIRT2 by Western blot after overexpression. **, significant difference (*p* < 0.01). The results were obtained in three independent experiments.

**Figure 4:**
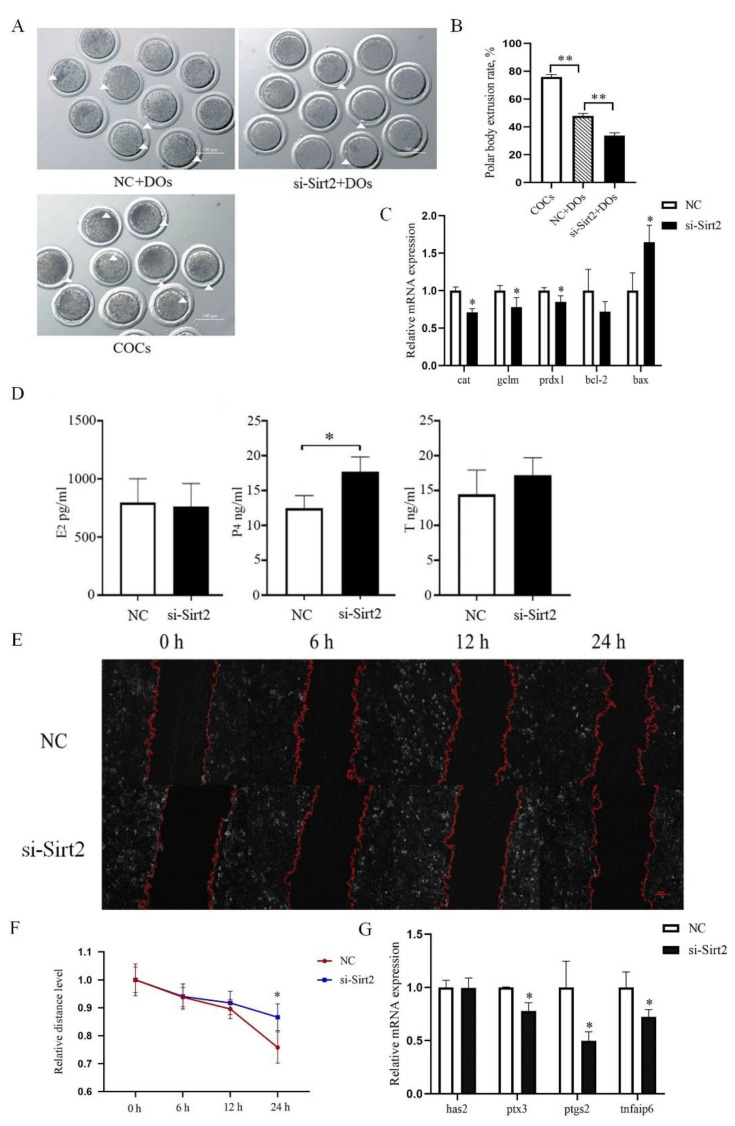
Sirt2 of granulosa cells is critical for sheep oocyte maturation and granulosa cell migration. (**A**) Representative images of oocyte maturation after Sirt2 knockdown in granulosa cells. The white triangle represents the extrusion of the polar body. Bar = 100 μm. (**B**) Oocyte maturation rate after Sirt2 knockdown in granulosa cells. (**C**) qRT-PCR detected the expression of oxidative and apoptotic genes in granulosa cells after Sirt2 knockdown. (**D**) The levels of E_2_, P_4_, and T in SIRT2-inhibited granulosa cells detected by ELISA. (**E**) Images showing scratch widths at 0, 6, 12, and 24 h after Sirt2 knockdown. Bar = 100 μm. (**F**) The granulosa cells migration distance compared between Sirt2 knockdown and control groups after 0, 6, 12, and 24 h. (**G**) The mRNA expression levels of granulosa cell expansion genes including has2, ptx3, ptgs2, and tnfaip6. The graph shows the mean ± SEM of the results obtained in three independent experiments. *, significant difference (*p* < 0.05). **, significant difference (*p* < 0.01).

The interaction between granulosa cell and oocyte regulates its maturation, so we intend to explore the function of granulosa cell during oocyte maturation process. To determine the knockdown efficiency in our method, as shown in Figure 3, the siRNA transfection sufficiently inhibited abundance of Sirt2 in sheep granulosa cells, and these cells were used for further study. The oocyte in vitro maturation rate significantly decreased after being cocultured with Sirt2-knockdown granulosa cells (47.98% ± 1.43 vs. 33.60% ± 1.77, *p* < 0.05, Figure 4A,B).

To further explore the functional mechanism of Sirt2 in granulosa cells, we detected its effect on oxidative apoptotic genes. The results showed that Sirt2 knockdown significantly decreased the expression of antioxidant genes including cat, gclm, and prdx1, and promoted the expression of pro-apoptotic gene (*p* < 0.05, Figure 4C). As steroid hormone secretion is an important aspect for oocyte maturation, we detected the secretion of E_2_, P_4_, and T after Sirt2 knockdown in granulosa cells. The secretion of P_4_ significantly increased in the si-Sirt2 group compared with the control (*p* < 0.05, Figure 4D), although the E_2_ and T secretion remain unchanged (*p* > 0.05, Figure 4D).

Next, cell migration and expansion-related gene expression of granulosa cells were also examined. Results of the scratch experiments showed that the width of scratches increased significantly after Sirt2 knockdown (*p* < 0.05, Figure 4E,F). Correspondingly, the mRNA abundance of expansion marker genes ptx3, ptgs2, and tnfaip6 were significantly lower in the Sirt2-knockdown group (*p* < 0.05, Figure 4G). These data suggest that Sirt2 plays a critical role in sheep oocyte in vitro maturation by affecting the apoptosis, expansion, and secretion function of granulosa cells.

### 2.3. Sirt2 Is Required for the Dynamic Balance of Mitochondrial Fusion and Fission and the Mitochondrial Quality

**Figure 5:**
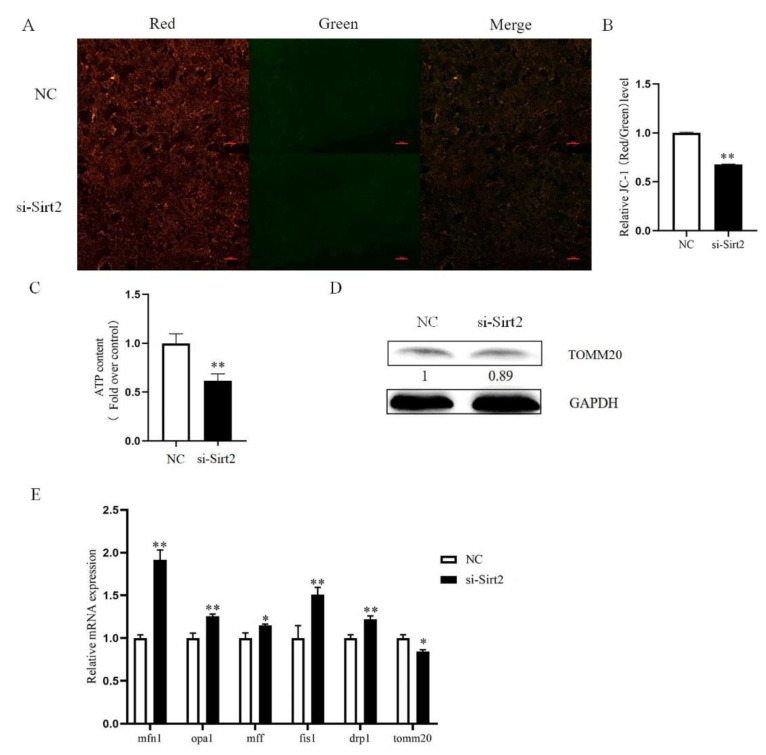
Sirt2 knockdown in granulosa cells affects mitochondrial function. (**A**) Images of red and green fluorescence in the NC and Sirt2 knockdown groups after JC-1 treatment. Bar = 100 μm. (**B**) Relative levels of mitochondrial membrane potential after Sirt2 knockdown. (**C**) ATP relative levels after Sirt2 knockdown. (**D**) Western blot detected the protein level of mitochondrial protein TOMM20 after Sirt2 knockdown. (**E**) The expression of granulosa cell mitochondrial genes was detected by qRT-PCR after Sirt2 knockdown. The graph shows the mean ± SEM of the results obtained in three independent experiments. *, significant difference (*p* < 0.05). **, significant difference (*p* < 0.01).

Mitochondrial membrane potential and ATP production are indicators of mitochondrial function. To explore the function of SIRT2 on mitochondrial of granulosa cells, we tested its mitochondrial membrane potential level and ATP production. Our results indicated that the red/green fluorescence ratio of JC-1 and ATP content were both significantly decreased in the Sirt2 knockdown group (*p* < 0.01, Figure 5A–C). Next, to measure the mitochondrial quality, the amount of TOMM20 was tested by Western blot analysis, and the amount of TOMM20 decreased after Sirt2 knockdown (Figure 5D). Then, we further detected the expression of mitochondrial fusion and fission genes and found that SIRT2 knockdown significantly increased the expression of mitochondrial fusion genes *mfn1*, *opa1,* and mitochondrial fission genes *fis1* and *drp1* (*p* < 0.01, Figure 5E), and the mRNA abundance of *tomm20* was significantly reduced (*p* < 0.05, Figure 5E). These results indicate that Sirt2 is required for the dynamic balance of mitochondrial fusion and fission and mitochondrial quality.

### 2.4. Effect of Sirt2 Overexpression on Granulosa Cell Mitochondrial Function

**Figure 6:**
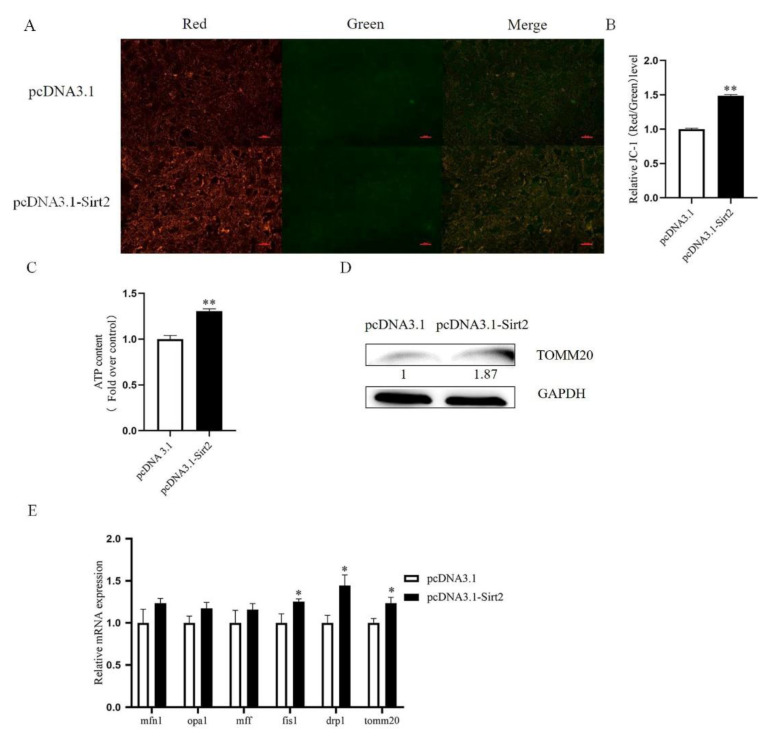
Overexpression of Sirt2 in granulosa cells affects mitochondrial function. (**A**) Mitochondrial membrane potential (red and green fluorescence) in the control group and Sirt2 overexpression group after JC-1 treatment. Bar = 100 μm. (**B**) Intensity of mitochondrial membrane potential after Sirt2 overexpression. (**C**) ATP relative levels after overexpression of Sirt2. (**D**) Western blot detected the protein level of mitochondrial protein TOMM20 after Sirt2 overexpression. (**E**) The expression of granulosa cell mitochondrial genes (mfn1, opa1, mff, fis1, drp1, and tomm20) was detected by qRT-PCR after overexpression of Sirt2. The graph shows the mean ± SEM of the results obtained in three independent experiments. *, significant difference (*p* < 0.05). **, significant difference (*p* < 0.01).

To further determine the gain-of-function effect of Sirt2 on mitochondrial function in sheep granulosa cells, the mitochondrial membrane potential, ATP production, and related gene expression were also investigated after Sirt2 overexpression. Results showed increased level of mitochondrial membrane potential and ATP content after its overexpression (*p* < 0.01, Figure 6A–C). The protein abundance of TOMM20 was also increased after Sirt2 overexpression (Figure 6D), suggesting an improved mitochondrial quality. We further detected the expression of mitochondrial fusion and fission genes and found that mitochondrial fission genes increased significantly after Sirt2 overexpression (*p* < 0.05, Figure 6E), indicating that mitochondrial division might be accelerated.

### 2.5. Sirt2 Knockdown Increases the Mitophagy Level

**Figure 7:**
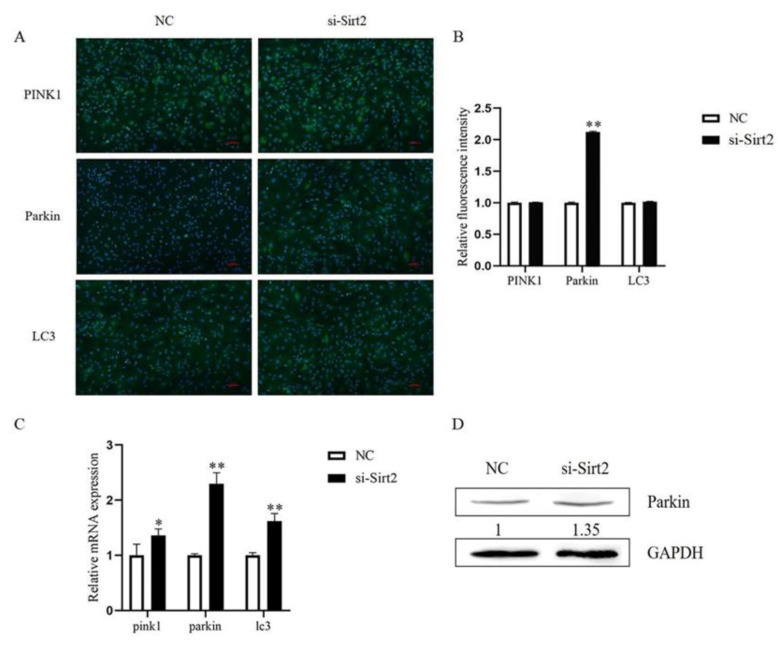
Sirt2 knockdown in granulosa cells affects mitophagy. (**A**) Immunofluorescence staining images of mitophagy proteins PINK1, Parkin, and LC3 after Sirt2 knockdown. Bar = 100 μm. (**B**) The relative expression level of mitophagy proteins by immunofluorescence staining after Sirt2 knockdown. (**C**) The mRNA relative expression level of mitophagy genes (pink1, parkin, and lc3) after Sirt2 knockdown. (**D**) The protein expression level of Parkin after Sirt2 knockdown. The graph shows the mean ± SEM of the results obtained in three independent experiments. *, significant difference (*p* < 0.05). **, significant difference (*p* < 0.01).

**Figure 8:**
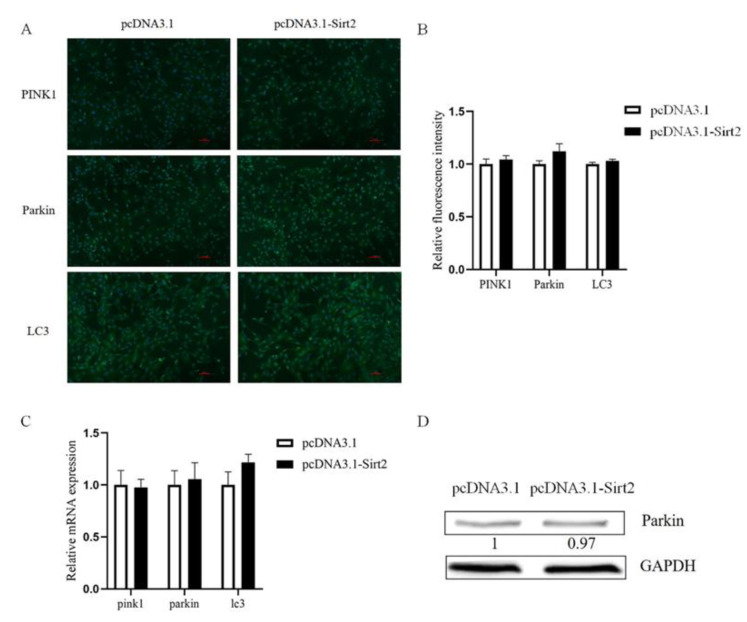
Overexpression of Sirt2 in granulosa cells had no effect on mitophagy. (**A**) Immunofluorescence staining images of mitophagy proteins PINK1, Parkin, and LC3 after Sirt2 overexpression. Bar = 100 μm. (**B**) The relative expression level of mitophagy proteins by immunofluorescence staining after Sirt2 overexpression. (**C**) The mRNA relative expression level of mitophagy genes (pink1, parkin, and lc3) after Sirt2 overexpression was detected by qRT-PCR. (**D**) The protein level of mitophagy protein Parkin after Sirt2 overexpression. The graph shows the mean ± SEM of the results obtained in three independent experiments.

As Sirt2 knockdown caused mitochondrial dysfunction, we tried to determine whether it had an impact on mitophagy. The immunofluorescence results showed a significant increase of Parkin in Sirt2 knockdown group (*p* < 0.01, Figure 7A,B), and the qPCR and Western blot analysis result showed that the mRNA and protein level of Parkin were both significantly increased (*p* < 0.01, Figure 7C,D). Surprisingly, no significant change was found in Parkin level after Sirt2 overexpressed (*p* > 0.05, Figure 8).

### 2.6. SIRT2 Regulates Steroid Hormones Secretion through ERK1/2 Signaling Pathway in Granulosa Cell

**Figure 9:**
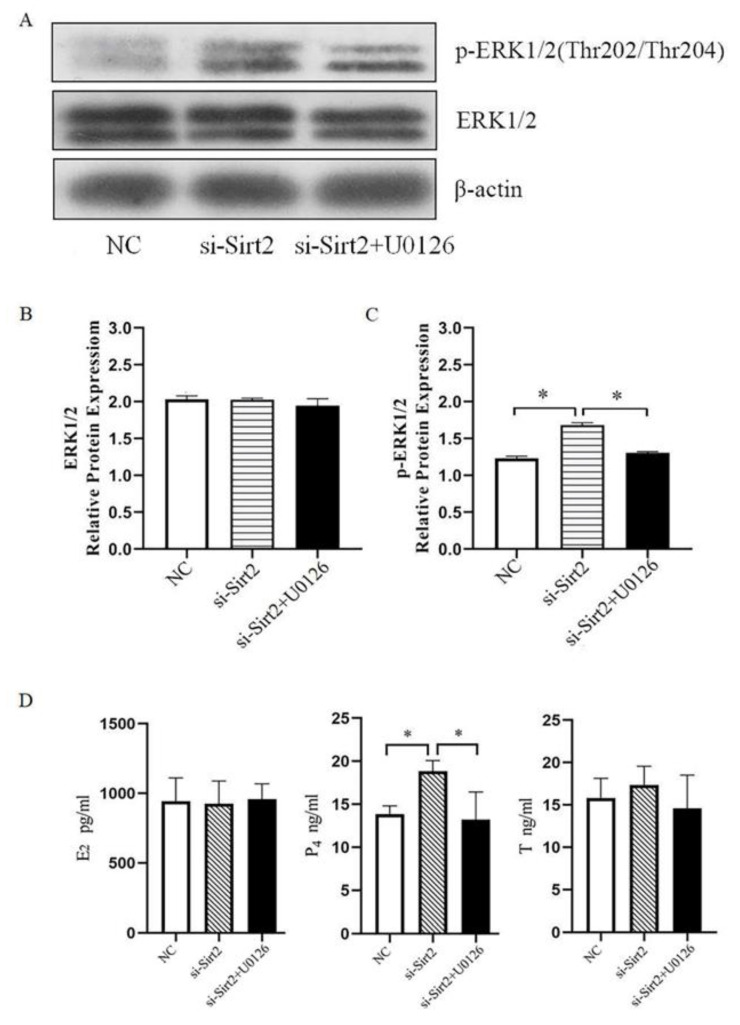
Sirt2 knockdown in granulosa cells affects steroid hormone secretion through ERK1/2. (**A**) Western blot representative images of p-ERK1/2 and ERK1/2 in different treatment groups. (**B**) ERK1/2 protein expression levels in different treatment groups. (**C**) p-ERK1/2 protein expression levels in different treatment groups. (**D**) The levels of E_2_, P_4_, and T secreted by granulosa cells in different treatment groups were detected by ELISA. The graph shows the mean ± SEM of the results obtained in three independent experiments. *, significant difference (*p* < 0.05).

To further explore the mechanism of effect of Sirt2 on steroid hormones secretion, we tried to determine whether Sirt2 influenced the ERK1/2 pathway, which showed involvement in the steroid hormone secretion process [15]. Western blot results showed that Sirt2 knockdown had no significant effect on protein level of ERK1/2, but it activated protein of p-ERK1/2. However, on the basis of Sirt2 knockdown, treatment with U0126, an ERK1/2 pathway inhibitor, significantly reduced the expression of p-ERK1/2 (*p* < 0.05, Figure 9A–C), indicating that Sirt2 is involved in the regulation of ERK1/2 signaling pathway. To further prove our hypothesis, we tested whether Sirt2 affects steroid hormone secretion through regulating the ERK1/2 pathway. The results showed that U0126 treatment could compensate for the increase of P_4_ caused by Sirt2 knockdown (*p* < 0.05, Figure 9D). These results indicate that Sirt2 can regulate the secretion of P_4_ through ERK1/2 pathway.

## 3. Discussion

Sheep granulosa cells, as an important part of follicles, are closely related to the maturation of oocytes and can secrete glucose metabolites, amino acids, and other nutrients to oocytes to promote oocyte maturation [2,3,16]. The effect of SIRT2 on the gap junction communication between bovine cumulus cells and oocytes has been proved by previous study [14]. In the present research, we focus on the role of SIRT2 during sheep oocyte maturation and its specific functional mechanism.

To confirm the role of Sirt2 in sheep oocyte maturation. we firstly inhibited SIRT2 in sheep COCs with supplementation of SIRT2 inhibitor and found reduced maturation rate and antioxidant capacity, which is consistent with results in mice and cattle [17,18], while the maturation rate and antioxidant capacity of sheep oocytes increased with resveratrol, a SIRT2 activator, in the culture medium, confirming the role of SIRT2 in sheep oocyte in vitro maturation. In order to further explore the mechanism of SIRT2 affecting oocyte maturation, we performed knockdown of Sirt2 in sheep granulosa cells and then cocultured with oocytes; the maturation rate of oocytes was also significantly decreased, indicating that Sirt2 in sheep granulosa cells is required for the maturation of sheep oocytes. The effects of Sirt2 on granulosa cells could be multi-sided, as Sirt2 is involved in the regulation of antioxidant, energy supply, and other physiological functions [19,20]. We found that the expression of antioxidant genes decreased and the expression of apoptotic genes increased after SIRT2 knockdown in granulosa cells, which is in agreement with previous reports confirming that upregulation of SIRT2 alleviates diabetic osteoarthritis development by suppressing oxidative stress [21]. The expansion of granulosa cells is involved in the development and maturation of oocytes [22,23]. In our study, Sirt2 knockdown affected granulosa cell migration, which is consistent with the result of granulosa cell expansion marker genes downregulation, such as *ptx3*, *ptgs2*, and *tnfaip6*. These findings suggested a potential regulatory role of Sirt2 in granulosa cell expansion, which affects the granulosa cell–oocyte microenvironment.

Mitochondria are interconnecting and highly dynamic cellular organelles whose homeostasis requires a stable equilibrium in a multitude of processes including fission, fusion, and biogenesis [24]. The relative balance of mitochondrial fission and fusion is crucial for maintaining mitochondrial quality and function, and abnormal expression of mitochondrial fusion and fission genes lead to disturbed mitochondrial structure and function [25]. In our study, both mitochondrial fusion and fission genes were significantly elevated, which could be the cell protective mechanism [26]. Consistent with previous study, the present research suggests that low doses and chronic exposures of substances increase fusion and slightly increase fission [27], thereby strengthening the mitochondrial network to remove damaged components [28]. Normal mitochondrial function of granulosa cells is a necessary environment for oocyte mitosis. The decrease of mitochondrial membrane potential of granulosa cells affects the synchronization of oocyte nucleus and ooplasm maturation [29]. In addition, reduction of mitochondrial membrane potential disrupts ion homeostasis and reduces ATP level [30], which is consistent with our results. However, highly polarized mitochondria in granulosa cells are important ATP sources for mammalian oocytes, and the decrease of ATP level affects the oocytes quality [7].

Mitophagy is a mechanism for clearing damaged mitochondria, and the PINK1-Parkin pathway plays a critical role in mitochondrial quality control by triggering the mitophagy clearance of damaged mitochondria [31]. In accordance with other reports, the mitophagy response of mitochondrial injury is a pro-survival mechanism [32]. According to previous results in our lab, abnormal mitochondrial dynamic balance and reduced mitochondrial membrane potential trigger mitochondrial mitophagy. When mitochondria damage happens, the mitochondrial membrane potential depolarizes, and recruits Parkin in cytoplasm to bind to the damaged mitochondrial membrane surface, thus mediating the occurrence of mitophagy [33]. This is probably the rationale to explain the significant increase of Parkin protein after Sirt2 knockdown in granulosa cells in the present study.

The steroidal hormones secreted by sheep granulosa cells are also required for oocytes growth and development. In our study, Sirt2 knockdown significantly increased the secretion of P_4_, which was consistent with results obtained in bovine [34]. In addition, the P_4_ secretion increase may be one of the reasons for the decrease of oocyte maturation rate, since addition of P_4_ to maturation medium leads to oocyte meiosis arrest [35]. ERK1/2 signaling pathway is involved in regulation of reproductive physiological functions, which plays a key role in regulating the steroid hormone secretion [36,37]. In this experiment, SIRT2 knockdown in granulosa cells increased the protein expression of p-ERK1/2 and activated the ERK1/2 signaling pathway, which is consistent with the result that p-ERK1/2 expression was increased after SIRT2 inhibition in bovine granulosa cells [14], while the use of U0126, an ERK1/2 inhibitor, reversed the upregulation of P_4_ secretion after SIRT2 inhibition. These results suggest that SIRT2 affects secretion of P_4_ in steroid hormones by regulating the ERK1/2 signaling pathway.

## 4. Materials and Methods

### 4.1. Ethics Statement

The experimental procedures were carried out in full accordance with the Guide for Care and Use of Agricultural Animals in Agricultural Research and Teaching. In addition, all experimental procedures were approved by the Animal Use Committee, Hebei Agricultural University.

### 4.2. Chemicals and Reagents

All chemicals and reagents were purchased from Sigma-Aldrich (St. Louis, MO, USA) unless otherwise stated.

### 4.3. Isolation and Culture of Granulosa Cells and Oocytes

Sheep ovaries obtained from a local slaughterhouse (Baoding, China) were placed in saline at about 37 °C and transported to the laboratory within 3 h. After washing the ovaries with saline, follicles from 3 to 6 mm in diameter were sliced, and cumulus–oocyte complexes (COCs) were obtained from the drained off follicular fluid. Then, the follicular fluid was collected into saline in a sterile environment, centrifuged, and cells were suspended in complete medium. Subsequently, granulosa cells were cultured in DME/F-12 medium (Corning, NY, USA) supplemented with 10% fetal bovine serum (FBS, Gibco, Carlsbad, CA, USA) at 38.5 °C in a humidified atmosphere of 95% air and 5% CO_2_. As for COCs, only those with three or more layers of cumulus and uniform ooplasm were selected, and then they were cultured in TCM199 supplemented with 10% FBS, 10 mg/mL FSH, 10 mg/mL LH, 1 mg/mL E_2_, 1 mmol/L L-glutamine, and 20 ng/mL EGF for 24 h at 38.5 °C and 5% CO_2_ in humidified atmosphere.

### 4.4. RNA Interference

A total of 1 × 10^5^ cells were seeded into 6-well plates containing cell culture medium. Both the SIRT2 siRNA (si-Sirt2) and the negative control siRNA (NC) were synthesized by Sangon Biotech (Shanghai, China). The transient transfection of granulosa cells with siRNA (20 μM) was performed for 24 h using Lipofectamine™ RNAiMAX transfection reagent (Invitrogen, Carlsbad, CA, USA) according to the manufacturer’s instructions at a confluence of 70%. The specific sequences are shown in Table 1.

### 4.5. Gene Overexpression

The cDNA sequence of SIRT2 was synthesized based on its mRNA sequences, and the synthesized SIRT2 sequence was inserted into pcDNA3.1(−) vector to construct the recombinant pcDNA3.1(−)/SIRT2-overexpressing vector. Lipofectamine 3000 transfection reagent (Invitrogen, Carlsbad, CA, USA) was mixed with pcDNA3.1-Sirt2 or pcDNA3.1 at a 1:1 ratio (*v*/*v*) at room temperature for 20 min. Then, the cells were incubated in regular 10% FBS DME/F-12 with pcDNA3.1-Sirt2 or pcDNA3.1 transfection mixtures for 24 h according to the manufacturer’s instructions.

### 4.6. Granulosa Cells Were Cocultured with DOs

The density of granulosa cell suspension was adjusted to 1 × 10^5^ cells/mL, and 150 μL of cell suspension was placed in each well of the 96-well plates. After the cell reached a confluence of 70–80%, NC and si-Sirt2 transfection was performed; 24 h later, granulosa cell culture medium was discarded, and 150 μL in vitro maturation medium of oocyte was added, covered with mineral oil, and balanced overnight in advance in a carbon dioxide cell incubator. Then, DOs were cultured in pre-balanced medium for 24 h to calculate the polar body excretion rate.

### 4.7. Cell Scratch Test

Cells were seeded into 6-well plates; when the cells reached a confluence of 70–80%, a cell scraper was used to draw straight lines in the cell monolayer in each well. The plates were then washed 3 times with serum-free medium. The cells were then cultured with serum-free medium, and the width of each scratch was observed with an optical microscope (Olympus, Shibuya, Japan) after 0, 6, 12, and 24 h of culture in a 38.5 °C incubator.

### 4.8. Intracellular Reactive Oxygen Species (ROS) and Glutathione (GSH) Levels Assay

The IVM oocytes at the metaphase II stage were sampled 24 h after IVM for determination of their intracellular ROS and GSH levels. Briefly, 2′7′-dichlorodihydrofluorescein diacetate (H2DCFDA) and 4-chloromethyl-6,8-difluoro-7-hydroxycoumarin (Cell Tracker Blue; CMF2HC) were used to detect intracellular ROS as green fluorescence and GSH level as blue fluorescence, respectively. A total of 15–20 oocytes from each treatment group were incubated in a carbon dioxide cell incubator for 20 min in phosphate-buffered saline (PBS) containing 10 μM H2DCFDA and 10 μM Cell Tracker Blue. After incubation, oocytes were washed with PBS and placed into 10 μL droplets, and then the fluorescence was observed using a fluorescence microscope (Olympus).

### 4.9. Detection of Mitochondrial Membrane Potential

Mitochondrial membrane potential was measured according to the instructions provided with the JC-1 Kit (Beyotime, Shanghai, China). Briefly, oocytes and granulosa cells were cultured in the dark for 20 min in the presence of JC-1 at a concentration recommended by the manufacturer (1 μg/mL). Subsequently, the red and green fluorescence was observed at the same position under a fluorescence microscope (Olympus). Membrane potential was calculated as the ratio of red florescence, corresponding to strongly activated mitochondria (J-aggregates), to green fluorescence, corresponding to less-strongly activated mitochondria (J-monomers).

### 4.10. Immunofluorescence Staining

Cell slides were added to 6-well plates before cell seeding, and then, a total of 1 × 10^5^ cells were seeded on the slides with cell culture medium. When the cells reached a confluence of 70–80%, the 6-well plates were washed with PBS 3 times. Then, the cells were fixed with 4% paraformaldehyde for 20 min, washed 3 times with PBS, and permeabilized with 0.2% Triton X-100 in PBS for 30 min. Then, blocking with 3% BSA was performed for 1 h at room temperature, and polyclonal rabbit anti-PINK1 (1:200, #23274-1-AP; Proteintech, Wuhan, China), rabbit anti-Parkin (1:200, # bs-23687R; Bioss, Beijing, China), and rabbit anti-LC3 (1:200, #18725-1-AP; Proteintech) antibodies were applied, and the cells were incubated overnight at 4 °C. After washing, cells were incubated with an Alexa Fluor 488-conjugated secondary antibody (1:300, #A-11034; ThermoFisher Scientific, Waltham, MA, USA) for 1 h. Then, the plates were washed three time with PBS, and stained with 4′,6-diamidino-2-phenylindole (DAPI, Beyotime, Shanghai, China) for 6 min. Finally, images were obtained using a fluorescence microscope (Olympus).

### 4.11. RNA Extraction and Quantitative Real-Time Reverse-Transcription Polymerase Chain Reaction (qRT-PCR)

Total cellular RNA was extracted using RNA Simple total RNA extraction kit (TIANGEN, Beijing, China). The cDNA was synthesized according to the manufacturer’s instructions (TaKaRa, Japan). QRT-PCR with SYBR fluorescent dye (Biotium, Bay Area, CA, USA) was used to detect the mRNA expression levels of the genes in the cells, and the PCR conditions were as follows: 95 °C for 2 min followed by 40 cycles of 95 °C for 5 s and 60 °C for 30 s. Finally, gene expression was quantified using the 2^−ΔΔCt^ method, and the primers used to amplify each gene are listed in Table 1.

### 4.12. Western Blot

After treatment, the cells were washed with cold PBS and lysed in lysis buffer (Biotopped, Beijing, China) with the protease inhibitor (Biotopped). The cell lysates were placed on ice for 30 min, and then centrifuged at 12,000 rpm for 10 min at 4 °C to collect the supernatant. The protein concentrations in the supernatant were quantified using a BCA protein assay kit (Biomed, Beijing, China), and sample proteins were separated by SDS-PAGE and transferred onto an NC membrane (Biotopped). The membranes were blocked with 5% nonfat dry milk in Tris aminoethane tween (TBST) buffer (Biotopped) for 2 h and then incubated with primary antibodies: SIRT2 (1:500, #19655-1-AP; Proteintech), Tomm20 (1:500, #11802-1-AP; Proteintech), Parkin (1:500, #bs-23687R; Bioss), p-ERK1/2 (Thr202/Tyr204, 1:1000, #9101; CST, Danvers, MA, USA), ERK1/2 (1:1000, #9101; CST), GAPDH (1:10000, #A01020; Abbkine, CA, USA), and β-actin (1:1000, #ab8226; abcam, Cambridge, England, UK) at 4 °C overnight. Following washing with TBST, membranes were incubated with related horseradish-peroxidase-conjugated secondary antibodies at room temperature for 1 h. Membranes were then incubated in ECL reagents (Biotopped) and images were captured by a chemiluminescence imaging analyzer.

### 4.13. ELISA

Granulosa cell culture medium of NC group and si-Sirt2 group were collected and centrifuged at 3000 rpm for 10 min, then supernatant was taken and stored in −80 °C. The concentrations of E_2_, P_4_, and T were determined by ELISA kits (Yaanda, Beijing, China).

### 4.14. Determination of Intracellular ATP

ATP detection kit (Beyotime, China) was used to evaluate ATP concentration of granulosa cells. Briefly, the culture medium was removed and a well of 6-well plate was lysed with 200 μL of cell lysis reagent. According to the kit’s instructions, 100 μL ATP working solution were added to 20 μL of sample and the standard solutions (0.01, 0.03, 0.1, 0.3, 1, 3, and 10 μM of ATP) in the 1.5 mL centrifuge tube, respectively. Subsequently, luminescence intensity was immediately measured using the Modulus™ Single tube multifunctional detector (Promega, Madison, WI, USA). A seven-point standard curve (0.01, 0.03, 0.1, 0.3, 1, 3, and 10 μM of ATP) was obtained for each series of analyses. Finally, the ATP concentration of the samples was calculated using the formula derived from the standard curve.

### 4.15. Statistical Analysis

All experiments were repeated at least three times. Data were shown as mean ± standard error of the mean (SEM). The results were analyzed by Student’s t-test or one-way ANOVA. ImageJ (NIH, Bethesda, MD, USA) was used to analyze scratch width, Western blot protein bands, and fluorescence images. *P* values < 0.05 were considered as significant using the SPSS 21.0 software (IBM Corporation, Armonk, NY, USA).

## 5. Conclusions

**Figure 10:**
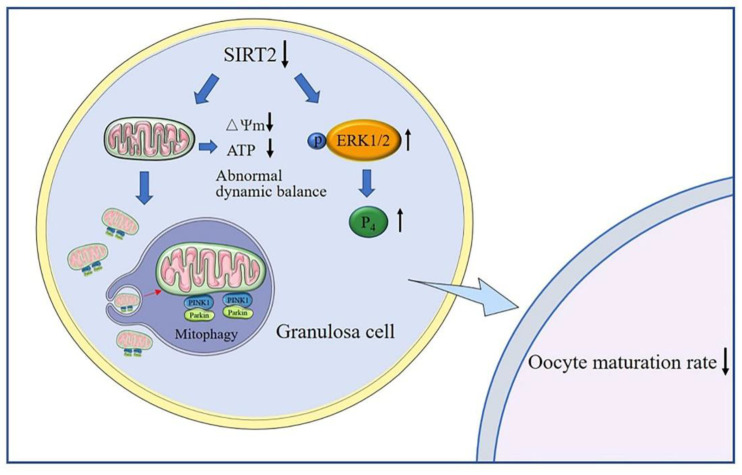
Mechanism of SIRT2 influencing sheep oocyte maturation through regulating surrounding granulosa cells. SIRT2 in granulosa cells is required for sheep oocyte maturation by affecting mitochondrial membrane potential and ATP level, mitophagy, and increasing the P_4_ secretion through regulating p-ERK1/2.

In the present study, inhibition or activation of SIRT2 in sheep COCs proved that it is critical for sheep oocyte maturation. Our study further indicated that SIRT2 is required in granulosa cells during the maturation of oocytes involved in the migration and diffusion ability, mitochondrial function, and steroid hormone secretion of granulosa cells (Figure 10).

## Figures and Tables

**Table 1:** Primers and siRNA used in this study.

Primers/siRNA Name	Primer Sequences (5’-3’)	Fragment Size (bp)
*β-actin*	F: CTCTTCCAGCCTTCCTTCCT R: GGGCAGTGATCTCTTTCTGC	178
*sirt2*	F: CGCCAACCTGGAGAAATA R: ATGGTGGGCTTGAACTGC	129
*gclm*	F: CCTATTGAAGATGGAGTGAATC R: GCAGGAGGCAAGATTAACT	189
*prdx1*	F: CAGATGGTCAGTTCAAGGAT R: CAGGTGACAGAAGTGAGAAT	191
*nfe2l2*	F: CATCACCAGACCACTCAG R: GGACTTACAGGCACTTCTT	241
*bcl-2*	F: GATGACCGAGTACCTGAACCG R: GACAGCCAGGAGAAATCAAACA	120
*bax*	F: CCGACGGCAACTTCAACTGG R: GATCAACTCGGGCACCTTGG	98
*c-myc*	F: TTGATGTTGTCTCTGTGGAA R: AATTGTGCTGATGCGTAGA	141
*has2*	F: CCTCATCATCCAAAGCCTG R: ACATTTCCGCAAATAGTCTG	139
*ptx3*	F: GCTATCGGTCCATAATGCTTG R: TTTCTTTGAATCCCAGGTGC	113
*ptgs2*	F: AGGAGGTCTTTGGTCTGGTG R: TCTGGAACAACTGCTCATCG	126
*tnfaip6*	F: CTACTGGCACATTAGACTCA R: AGCATCACTTAGGAACTTCA	217
*mfn1*	F: AAGCACATAGAAGACGGAAT R: ACGATGGACAAGAGAAGAC	264
*opa1*	F: ATGAAATAGAACTCCGAATG R: GTCAACAAGCACCATCCT	112
*mff*	F: GTGCTTACGCTGAGTGAA R: ACGAGTGGAAGACTGGATA	354
*fis1*	F: GGGGAACTACAGGCTCAAG R: GACACAGCAAGTCCGATGA	207
*drp1*	F: GGAGTTGAAGCAGAAGAATG R: AGTGACAGCGAGGATAATG	306
*tomm20*	F: GGACCATCTGACGAATGC R: GAGCACTTACAATTCTCTGAC	140
*pink1*	F: CCTGAAGTCCGACAACAT R: AGCCAATCATCTCGTCTG	266
*parkin*	F: CTTCATCATCAACCAGTTCTC R: CTTCAGCACAGGAATCAGT	371
*lc3*	F: TATCCGAGAGCAGCATCC R: GATCAGGCACCAAGAACTT	104
si-Sirt2	Sense: UCUUGAAGUAGCUGAUUUCAA Antisense: GAAAUCAGCUACUUCAAGAAG	
NC	Sense: UUCUCCGAACGUGUCACGUTT Antisense: ACGUGACACGUUCGGAGAATT	

## Data Availability

Not applicable.
